# Functional role and folding properties of the glucan‐binding domain of oral bacterial glucansucrase

**DOI:** 10.1002/1873-3468.70128

**Published:** 2025-08-02

**Authors:** Hideyuki Komatsu, Takayuki Sadakane, Yudai Murata, Junichi Taira, Hiroshi Sakamoto, Takao Kodama

**Affiliations:** ^1^ Department of Bioscience and Bioinformatics, School of Computer Science and Systems Engineering Kyushu Institute of Technology Fukuoka Japan

**Keywords:** cooperative unfolding, dextran, glucansucrase, interdomain allostery, protein–polysaccharide interaction

## Abstract

A *Streptococcus sobrinus* glucansucrase (i.e., glucosyltransferase‐I) elongates α‐1, 3‐linked glucose chains at pre‐existing branch points of dextran, and its activity is enhanced by primer dextran. The glucansucrase contains a catalytic domain and a glucan‐binding domain (GBd) with six tandem repeats. We here examined the role of GBd in the glucansucrase, focusing on its impact on enzymatic activity, dextran binding, and structural stability. By generating seven deletion mutants and a circularly permuted protein, our research demonstrates that the first four tandem repeats in the GBd and proper domain orientation are required for efficient glucosyl transfer. Moreover, characterization of circular dichroism and thermal unfolding indicated that glucosyl transfer efficiency is linked to cooperative interdomain folding. These findings highlight the importance of interdomain allostery in optimizing glucansucrase function.

**Impact statement**

Our research on the role of the glucan‐binding domain in *Streptococcus sobrinus* glucansucrase revealed that glucosyl transfer efficiency is linked to cooperative interdomain folding. The finding highlights the importance of interdomain allostery in optimizing glucansucrase function and also suggests potential targets for inhibiting bacterial biofilm formation.

## Graphical abstract

The role of the glucan‐binding domain in *Streptococcus sobrinus* glucansucrase was examined, focusing on its impact on enzymatic activity, dextran binding, and structural stability of deletion mutants and a circularly permuted protein. Our research revealed that glucosyl transfer efficiency is linked to cooperative interdomain folding. These findings highlight the importance of interdomain allostery in optimizing glucansucrase function and suggest potential targets for inhibiting bacterial biofilm formation.

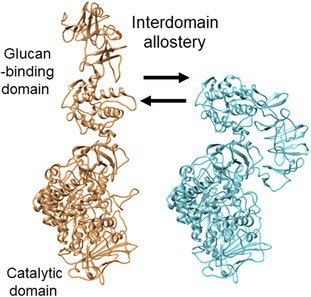

## Abbreviations

**CD**, circular dichroism

**DEAE**, diethylaminoethyl

**DSR**, dextransucrase

**GBd**, glucan‐binding domain

**GSd**, glucansucrase domain

**GTF**, glucosyltransferase

**ITC**, isothermal titration calorimetry

***K***_**d**_, dissociation constant

**MOPS**, 3‐(N‐morpholino)propanesulfonic acid

**PCR**, Polymerase chain reaction.

**SDS/PAGE**, Sodium dodecyl sulfate polyacrylamide gel electrophoresis

***T***_**m**_, transition temperature

**Tris**, tris(hydroxymethyl)aminomethane

**UV**, ultraviolet

**YT**, yeast extract tryptone

**Δ*G***, Gibbs energy change

**Δ*H***, enthalpy change

**Δ*S***, entropy change

Glucansucrases (EC 2.4.1.5; glucosyltransferases; GTFs) are extracellular enzymes produced by lactic acid bacteria that catalyze the transfer of glucosyl moieties from sucrose to create long α‐glucan chains [1]. Many α‐glucans produced by lactic acid bacterial glucansucrase are used as food additives, as medicines, and in biochemical research [2]. However, many of the product polysaccharides synthesized by mutant streptococci enhance colonization by cariogenic bacteria, thereby promoting the formation of dental plaque on the smooth tooth surface [3, 4]. Thus, these enzymes are linked to the development of dental caries [5].

Multiple sequence alignment of glucansucrase proteins revealed that each glucansucrase generally comprises a signal peptide, an N‐terminal variable region that includes a glucan‐binding domain, a catalytic domain (i.e., the glucansucrase domain, GSd), and a C‐terminal glucan‐binding domain (GBd) [1, 6, 7]. Based on the alignment of the catalytic core of the GSd [8], glucansucrases are classified as members of the glycoside hydrolase 70 (GH70) family. Furthermore, homology among Streptococcal GTFs, *Leuconostoc* glucansucrase, and GTFs of *Lactobacillus* suggests that the genes encoding these enzymes share common ancestry [9, 10].

The crystal structures of four truncated forms of glucansucrase (i.e., *Lactobacillus reuteri* 180 GTF180 [11], *L. reuteri* 121 GTFA [12], *Streptococcus mutans* GTF‐SI [13], and *L. mesenteroides* DSR [14, 15]) reveal that these enzymes have a common domain architecture in their catalytic cores, which contains domains A, B, and C as well as domain IV [10, 11]. Notably, domains A, B, and IV are each composed of two discontinuous polypeptide chains, and therefore, the polypeptide chain as a whole adopts a U‐shaped form. On the basis of their structures of the catalytic core, mechanisms of reaction and linkage specificity have been studied [16, 17, 18]. In contrast, the GBd within glucansucrase is composed of a series of homologous tandem repeats, known as the A, B, C, and D repeats [19, 20]. Crystal‐based structural analyses of these motifs, including the GBd (domain V) of glucansucrase, demonstrated that these motifs form a β‐solenoid fold containing 4 units, each consisting of a β‐hairpin or triple‐stranded antiparallel β‐sheet [10, 15].

Continuous studies have indicated that the deletion of a repeating unit at the C‐terminal end of streptococci and lactic acid bacteria glucansucrase resulted in strongly reduced GTF activity, which suggests that the glucansucrase GBd accelerates glucosyl transfer for products at the catalytic site [6, 21, 22, 23, 24, 25, 26, 27, 28, 29]. Indeed, further study has revealed two types of crystal structures present in the *L. mesenteroides* DSR‐E, known as ‘straight’ and ‘bending’ forms. Their identification suggests that the swinging motion of the GBd moves growing glucan products close to the active site [14, 15]. In addition, a small‐angle X‐ray scattering analysis demonstrated this swinging motion of *Lactobacillus reuteri* GTF180 in solution [30]. Therefore, swinging motion between the catalytic domain and the GBd bound to dextran or another glucan likely occurs during glucosyltransfer [2, 15, 30]. However, to date it remains unclear whether enhanced glucosyltransfer activity is caused by interdomain allostery or mere proximity effects between dextran and the catalytic site. Moreover, it is also unknown how loss of hinge movement actually affects GS activity. Thus, important questions regarding the role of the glucansucrase GBd remain unanswered.

In a previous study, our group used isothermal titration calorimetry to find that the tandem repeats within the GBd of *Streptococcus sobrinus* 6715 GS play a role in dextran binding [31]. Moreover, we also demonstrated that there was a linear relationship between the number of repeats and binding affinity, and thus the strong binding of GBd to dextran was facilitated by the multiple binding sites of the GBd. Further kinetic investigation of glucan synthesis via the *Streptococcus sobrinus* GS indicated that glucan synthesis and linkage specificity are both determined by the catalytic domain alone [32]. This result suggests that the GBd can increase the efficiency of catalysis and the specificity of the acceptor glucan during glucan synthesis. When combined with the findings of related studies, the coordination of the catalytic domain, hinge region, and GBd together appears to be essential for effective glucosyltransfer.

**Fig. 1:**
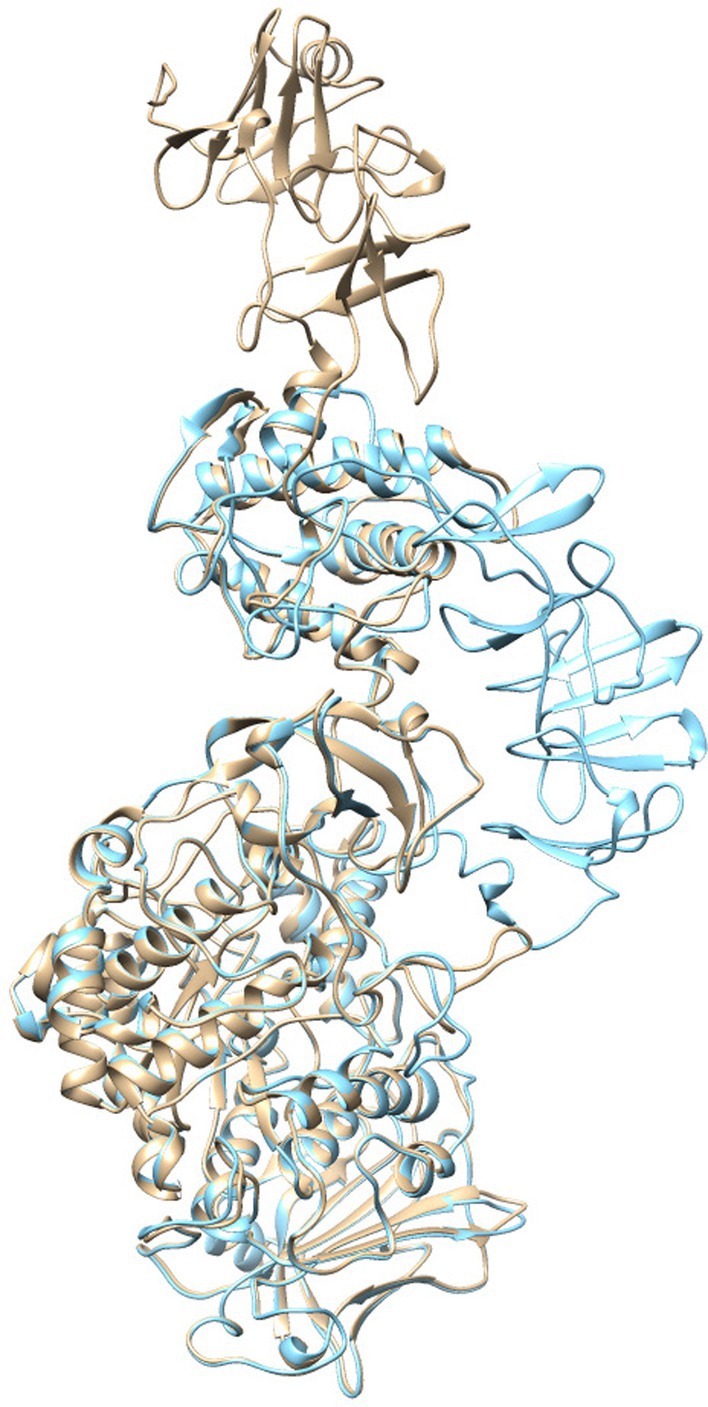
Homology modeling of glucosyltransferase (GTF)‐I based on the *Lactobacillus* GTF‐180 crystal structure. The straight and bending structures of *Streptococcus sobrinus* GTF‐I are indicated in beige and sky blue, respectively. The straight and bending models are modeled based on straight and bending conformations (PDB ID codes 3KLK and 4AYG) of *Lactobacillus* GTF‐180, respectively, in the SWISS‐MODEL server [34].

**Fig. 2:**
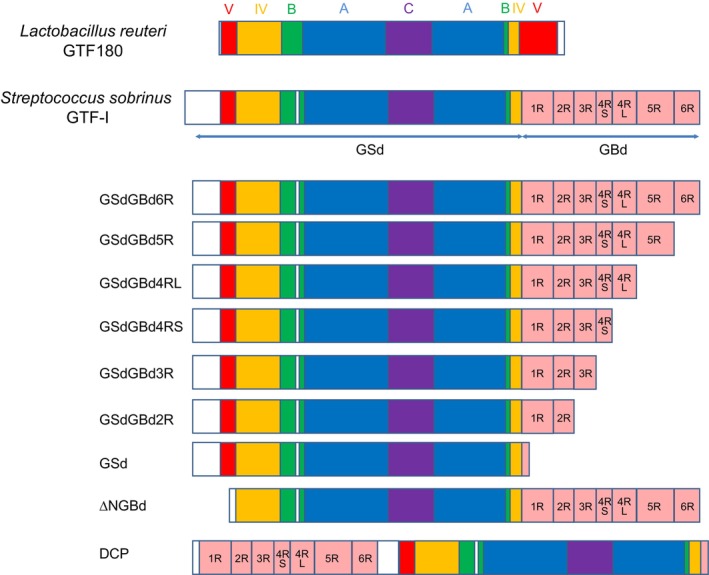
Schematic structures of glucosyltransferase (GTF)‐I and its mutant proteins. Subdomains V, IV, B, A, and C of the crystal structure of *Lactobacillus reuteri* GTF180 are colored as red, yellow, green, blue, and purple. Next, subdomains of the glucansucrase domain (GSd) of *Streptococcus sobrinus* GTF‐I and its mutant proteins were determined via residue sequence alignment. The glucan‐binding domain (GBd) is indicated in pink and is divided into 6‐repeat sequences, including conserved glycine and aromatic residues that are characterized as a glucan‐binding motif. The bottom structure indicates a domain‐circular permutated protein (DCP) between GSd and GBd. Short peptides (6, 10, or 11 residues) derived from the multi‐cloning site of pUC18 are attached at the N‐terminal end.

Here, to investigate the role that GBd plays in regulating the activity of *Streptococcus sobrinus* GS, we first constructed homology models of both the straight and bending forms of *Streptococcus sobrinus* GS. This was performed as per the configuration suggested by the crystal structure of *L. mesenteroides* DSR‐E [14, 15] (Fig. 1), since the structure of the GBd‐linked catalytic domain of the streptococci GS remains unelucidated. These models suggest that the *S. sobrinus* GS can also have straight and bending forms and can ‘swing’ the GBd in relation to the catalytic domain. On the basis of these homology models, we designed deletion and permutated mutant proteins to study the role of GBd on streptococci GS activity (Fig. 2). First, we examined the effect of the length of the GBd by using a series of truncation mutants, which are named based on the number of A repeats (e.g., GSdGBd2R represents a protein composed of GSd with 1st and 2nd A repeats of GBd, and additionally GSdGBd4RS and GSdGBd4RL are termed as shorter and longer polypeptide of GSdGBd4R, respectively). Throughout this study, GSdGBd6R was considered to be the wild‐type GS since it contains the full‐length GBd, lacking only 84 N‐terminal residues that contain the sequence of a 38‐residue signal peptide. Second, we produced other mutants in which the N‐terminal GBd domain was deleted (called ‘ΔNGBd’). Due to the U‐shaped form of the polypeptide chain, a portion of the GBd exists at the N terminus of the catalytic domain. This N‐terminal part of the GBd is also predicted to act as part of the hinge region since it is positioned as part of the linkage between the catalytic domain and the GBd. Further analysis of this mutant may provide additional information regarding the nature of the swing motion. Third, we constructed a domain‐circular permutated protein (DCP) between two domains (i.e., GSd and GBd) to test whether its activity was enhanced via proper orientation of GBd to GSd or by proximity effects of the acceptor bound to GBd (Fig. 2).

Finally, in this study, these mutant proteins were analyzed for glucansucrase activity, dextran‐binding affinity, structural characterization, and thermal unfolding cooperativity. Overall, we found that domain cooperativity was essential for enhanced glucosyltransfer activity to dextran.

## Materials and methods

### Homology modeling

Amino acid sequences (accession numbers are in parentheses) were aligned between *Streptococcus sobrinus* GTF‐I (D90213) and *Lactobacillus* GTF‐180 (AY697430) using Clustal W [33] to identify the homologous sequence. Straight and bending models of *Streptococcus sobrinus* GTF‐I were built by homology modeling as structural templates of straight and bending conformations (PDB ID codes 3KLK and 4AYG) of *Lactobacillus* GTF‐180, respectively, in the SWISS‐MODEL server [34].

### Plasmid construction

Plasmids pGS [24] and pGBD6R [31] were used to express the N‐terminal catalytic domain (GS; amino acid 85–1084) and the C‐terminal glucan‐binding domain (GBd; amino acid 1084–1592), respectively. Furthermore, plasmids pGS‐GBD6R [32] and pAB2 [23] were used to express GSdGBd6R and GSdGBd4RS, respectively. Plasmids pGS‐GBD6RdH, pGBD5R, pGBD4RL, pGBD3R, and pGBD2R were used to construct plasmids encoding GSdGBd5R, GSdGBd4RL, GSdGBd3R, and GSdGBd2R, respectively. Each of the previously reported plasmids pGBD5R, pGBD4RL, pGBD3R, and pGBD2R contains a stop codon at the start of each ‘A’ repeat on pGBD6R, which encodes the full‐length GBd (i.e., amino acids 1084–1592 of the GTF‐I sequence) [31]. pGS‐GBD6RdH was prepared by erasing a *HindIII* site (position 1946 bp within the GTF‐I gene) of pGS‐GBD6R via site‐directed mutagenesis (AAGCTT → AAGCCT) implemented using a Quick Change™ Site‐Directed Mutagenesis Kit (STRATAGENE). The primers used for mutagenesis were as follows: 5′ end primer, 5′‐CCCAAGAAGAAATTGATCAAGCCTTCAAG and 3′ end primer, 5′‐CTTGAAGGCTTGATCAATTTCTTCTTGGG. Next, plasmids pGBD5R, pGBD4RL, pGBD3R, and pGBD2R were all digested with *HindIII*. At the same time, pGS‐GBD6RdH was digested with *HindIII* and its GBd encoding region was removed via gel electrophoresis extraction, after which the regions encoding GBd in each plasmid were inserted at the *HindIII* site. This site corresponds to the end of the region encoding GSd (i.e., position 3432 bp of the GTF‐I gene of *S. sobrinus* 6715; [21]) within the pGS‐GBD6RdH fragment. The resulting plasmids were named pGS‐GBD5R, pGS‐GBD4RL, pGS‐GBD3R, and pGS‐GBD2R.

To construct pGBD6R‐GS, which encodes the domain‐circularly permuted protein (DCP), we first amplified a 1.5‐kb fragment encoding the full‐length GBd (i.e., amino acids 1084–1592) by PCR using the following primers: 5’‐AGCTTCATTACCGAAGCAG (5′ end) and 5′‐GTTCCAGCCACGGTAG (3′ end). The PCR product was inserted at the multiple cloning sites of pGS following digestion with *SmaI*. pGBD6R‐GS encodes amino acids 1084–1592 followed by amino acids 85–1084, with a single glycine added between them.

To construct pΔNGBDGS‐GBD6R, which encodes the N‐terminal glucan‐binding domain (amino acid 85–212)‐truncated GSdGBd6R (ΔNGBd), we PCR‐amplified a 1.2 kb fragment that encodes a part of GSd (amino acids 213–597) using the 5′ end primer, 5′‐AAAGAATTCCTTTGCAGCTAATAACCGT and the 3′ end primer, 5′‐GAAAAGATCTTCGTTGTAAATCTTGAAAGC. pGS‐GBD6R was then digested with *EcoRI* and *BglII*, and the *EcoRI*–*BglII* fragment (1.6 kb) was removed via gel electrophoresis. The PCR product was then inserted between the *EcoRI* site present in the multiple cloning site and the *BglII* site of *EcoRI*–*BglII*‐digested pGS‐GBD6R. Importantly, pΔNGBDGS‐GBD6R encodes amino acids 213–1592 of GTF‐I.

The following plasmids all encoded an extra peptide from pUC18 at the N‐terminal end: pGS, pAB2, pGS‐GBD6R, pGS‐GBD5R, pGS‐GBD4RL, pGS‐GBD3R, pGS‐GBD2R, TMITNSSSVPG; pGBD6R‐GS, TMITNSSSVP; pΔNGBDGS‐GBD6R, TMITNS; and pGBD6R, TMITNSSSVPGDPLESTCHR. All construction procedures are further summarized in Fig. S1.

### Protein expression and purification

For protein expression, we first expressed GSd, GSdGBd4RS, GBd6R, and GSdGBd6R in *E. coli* JM109 transformed with pGS, pAB2, pGBD6R, and pGS‐GBD6R, respectively and then performed protein purification using a previously described protocol [24, 31, 32].

Next, GSdGBd5R, GSdGBd4RL, GSdGBd3R, GSdGBd2R, and ΔNGBd were prepared from *E. coli* JM109 transformed with pGS‐GBD5R, pGS‐GBD4RL, pGS‐GBD3R, pGS‐GBD2R, and pΔNGBDGS‐GBD6R. Bacteria were grown at 37 °C in 2 × YT medium containing ampicillin (100 μg·mL^−1^). Protein expression was then induced by isopropyl‐β‐d‐thiogalactoside at an absorbance of 1.0 at 660 nm. After 3 h of incubation, cells were harvested by centrifugation, washed twice with 5 mm MgCl_2_, 5 mm KCl, and 10 mm Tris–HCl (pH 7.4), and then stored in a freezer (−20 °C) until further use. When it was time to conduct assays, frozen cells were suspended in 10 mm K‐phosphate (pH 6.8) before being treated with an ultrasonic disruptor (UD‐201; Tomy Seiko Co., Tokyo, Japan) for 5 min on ice. After removal of insoluble material by centrifugation, the supernatant was then concentrated via ammonium sulfate precipitation. Next, the precipitate was dialyzed against 10 mm K‐phosphate (pH 6.8) before being loaded onto a Toyopearl DEAE‐650M column (TOSOH) (2.5 × 20 cm) then eluted with 500 mL of a 0–0.4 m NaCl linear gradient. Moreover, GSdGBd3R (alone) was also purified using affinity chromatography [35]. To do so, a bacterial cell extract was fractionated with 30% ammonium sulfate, after which the supernatant was mixed with Sephacryl S‐300 beads (Pharmacia). The protein was then eluted with dextran (i.e., sourced from Leuconostoc ssp., Mr ~6000, Fluka) after washing with a buffer containing 10 mm K‐phosphate (pH 6.8). To remove dextran from the eluent, samples were chromatographed on a DEAE‐Toyopearl 650M and dialyzed against a 10 mm K‐phosphate buffer (pH 6.8). Finally, enzyme proteins were detected via SDS/PAGE and a sucrose‐hydrolysis assay using 3,5‐dinitrosalicylic acid [36].

Next, DCP was expressed in *E. coli* X‐L1Blue transformed with pGBDGS and grown at 37 °C in 2 × YT medium with ampicillin (100 μg·mL^−1^). After the cell density reached an absorbance of 1.0 at 660 nm, cells were harvested by centrifugation. Subsequently, DCP was extracted and purified as for GSdGBd5R, GSdGBd4RL, GSdGBd3R, GSdGBd2R, and ΔNGBd.

Protein purity was ascertained by SDS/PAGE (Fig. S2).

### Dextran and sucrose

Dextran T5 (dextran Standard 5000) and T25 (dextran Standard 25 000) were purchased from Fluka. The sucrose used for all experiments was of analytical grade. To remove trace amounts of high‐molecular‐weight glucose polymers, sucrose was first purified via centrifugal ultrafiltration using a 50 000‐molecular‐weight cutoff filter (Vivaspin 6 and 20 mL, Viva Science, Hannnover, Germany) and a 5000 Da exclusion filter (Centricon Plus‐20, Millipore, Bedford, MA, USA), respectively.

### Protein and carbohydrate concentrations

Protein concentrations were determined by recording sample absorbance at 280 nm using a molar extinction coefficient. This was calculated from deduced amino acid compositions [37]. Finally, dextran determination was performed using the phenol‐sulfuric acid method [38].

### Enzyme assay

Enzyme activity was measured using a previously described protocol [24]. Briefly, the enzyme activity reaction mixture contained 10 nm glucansucrase, 10 mm MOPS buffer adjusted to pH 7.0, 100 mm NaCl, 0–50 mm sucrose, and 0–1200 μg·mL^−1^ dextran T5 (i.e., Dextran from *Leuconostoc* ssp. Mr. ~5000, Fluka). All measurements were made at 25 °C after 4 min of reaction time.

### Isothermal titration calorimetry (ITC)

Isothermal titration experiments were carried out on an MCS‐ITC platform (MicroCal Inc., Northmpton, MA, USA) at 25 °C. First, protein samples were dialyzed against 10 mm K‐phosphate (pH 6.8), and dextran (i.e., a Dextran Standard 25 000 from *Leuconostoc mesenteroides*, Fluka) was dissolved into the dialysis buffer. An aliquot of the resulting solution was then injected once every 3 min into the protein solution; this was performed 20 times in total. The protein concentrations used were as follows: GSd, 15.9 μm; GSdGBd2R, 15.9 μm; GSdGBd3R, 7.66–36.9 μm; GSdGBd4RS, 7.62–14.2 μm; GSdGBd4RL, 16.9 μm; GSdGBd5RS, 11.8 μm; GSdGBd6R, 5.58–7.33 μm; DCP, 6.19 μm; and ΔNGBd, 2.69–4.00 μm. The dextran concentrations used were as follows: 14.2 mg·mL^−1^ for GSd, 14.2 mg·mL^−1^ for GSdGBd2R, 10.8–14.2 mg·mL^−1^ for GSdGBd3R, 1.95–3.13 mg·mL^−1^ for GSdGBd4RS, 1.77 mg·mL^−1^ for GSdGBd4RL, 1.57 mg·mL^−1^ for GSdGBd5R, 0.633–0.813 mg·mL^−1^ for GSdGBd6R, 0.726–0.782 mg·mL^−1^ for DCP, and 0.486 mg·mL^−1^ for ΔNGBd. For all experiments, exothermic **reaction** heat was measured by injecting the dextran solution into the dialysis buffer.

Next, all data were analyzed as per a previously reported protocol [31] with slight modification. Briefly, raw ITC data were first integrated and corrected for the mixing heat of dextran using Origin Lab software installed on the MCS‐ITC platform. Next, the specific heats for each titration step were then plotted as a function of the molar ratio of the glucose equivalent concentration of dextran to the total protein concentration. The titration curve thus obtained was then plotted as a nonlinear regression curve based on a model that assumed that GTF‐I recognizes and binds to a dextran stretch of *n* glucose units [31]. This regression was plotted using the data analysis suite present in Origin. We therefore obtained estimates of the number of glucose units constituting the dextran stretch *n*, the dissociation constant of a mutant glucansucrase for a dextran stretch of *n* glucose units *K*_d_, and the enthalpy change Δ*H*. Furthermore, other thermodynamic parameters (Gibbs energy change Δ*G* and entropy change Δ*S*) were derived from the equations Δ*G*° = *RT*ln*K*_d_ and Δ*G*° = Δ*H*°–*T*Δ*S*°, where *R* is the gas constant and *T* is the absolute temperature. The standard enthalpy change Δ*H*° is routinely approximated by the observed enthalpy change.

### Circular dichroism

Circular dichroism (CD) measurements were obtained using a J‐720 spectropolarimeter (JASCO). For the far‐UV region (i.e., 190–250 nm), the protein concentration was 1.6–2.6 μm and a path‐length of 0.1 cm was used, while for the near‐UV region (i.e., 250–320 nm), the protein concentration was 2.6–5.1 μm and a path‐length of 1 cm was used. Measurements were performed in 10 mM K‐phosphate, pH 6.8 at 20 °C. All spectral data were recorded at 0.1 nm decrements with a scan speed of 50 nm·min^−1^ and a bandwidth of 2.0 nm. Eight scans were performed on each sample and their values averaged. The observed ellipticities were then converted to mean residue ellipticities using the mean molecular mass per residue.

For thermal denaturation studies, the temperature was raised at a speed of 1 °C·min^−1^ using Peltier device‐type temperature controls (JASCO). Sixteen spectra were first recorded over a range from 36 to 66 °C at intervals of 2 °C. Next, temperature‐induced unfolding curves were monitored at 292 nm with intervals of 0.1 °C. The protein concentration was 2.3–5.4 μm, and other conditions were as described above. To analyze the mode of transition, we fitted temperature (*T*) *vs*. ellipticity (θ) curves using a nonlinear least‐squares protocol to obtain the midpoint unfolding temperature (*T*_m_). Eq. (1) was used for the single transition model [39]:

(1) $$\theta =\theta_{u}+\frac{\theta_{f}-\theta_{u}}{1+e^{\frac{T-T_{m}}{a}}}$$

Here, θ_u_ and θ_f_ are the molar ellipticities of the unfolded and folded proteins, respectively, and *a* is the curvature parameter. Next, Eq. (2) was used for the double‐transition model:

(2) $$\theta =\theta_{u}+\left(\theta_{f}-\theta_{u}\right)\left[\frac{F}{1+e^{\frac{T-T_{m1}}{a_{1}}}}+\frac{1-F}{1+e^{\frac{T-T_{m2}}{a_{2}}}}\right]$$

Here, *T*_m1_ and *T*_m2_ are the midpoint temperatures of the first and second transitions, and *a*_1_ and *a*_2_ are the curvature parameters of the first and second transitions, respectively. *F* represents the fraction of the first transition.

## Results

### Enzyme activities

Initially, in order to evaluate the GBd‐independent catalytic function of GSd of the truncated and permuted enzymes, activities were measured in 25 mM sucrose in the absence of primer dextran for the first 4 min of reaction time. In this condition, any transfer to sucrose, glucose, and fructose was not detected [24], and equal amounts of glucose and fructose were detected as hydrolyzing products. We found that the sucrose‐hydrolysis activity of all enzymes remained constant at 14–16 s^−1^ (data not shown). Accordingly, GSd of all enzymes retained the basal catalytic function of *S. sobrinus* GTF‐I [24, 32].

**Fig. 3:**
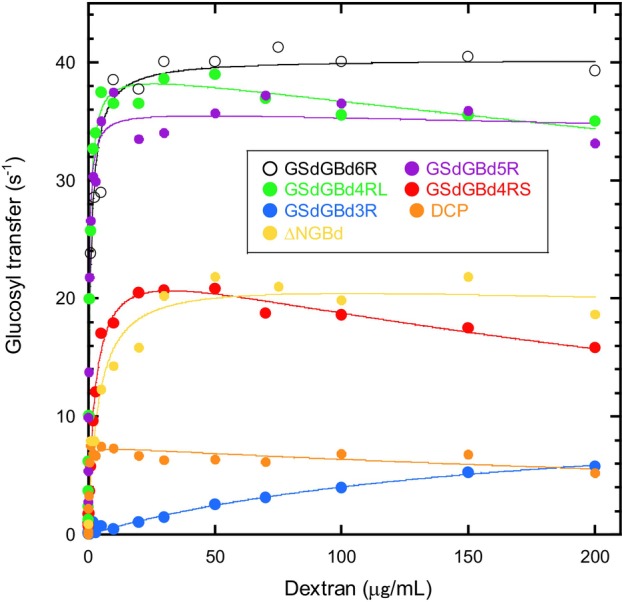
Glucosyl transfer activities to dextran of glucosyltransferase (GTF)‐I proteins. Shown are glucosyl transfer activities plotted against dextran concentration. GSdGBd6R, GSdGBd5R, GSdGBd4RL, GSdGBd4RS, GSdGBd3R, DCP, and ΔNGBd are indicated in white, purple, light green, red, blue, orange, and yellow, respectively. The data shown in the figure are representative of two independent experiments with similar results.

Figure 3 shows the glucosyl transfer activities of the mutant enzymes over a wide range of dextran concentrations. The activities were monitored under initial velocity assay conditions. We first note that GSdGBd2R and GSd did not show any dextran‐dependent glucosyl transfer activity at all (data not shown). Moreover, the glucosyl transfer activity of GSdGBd6R was saturated at approximately 40 s^−1^ for 50 μg·mL^−1^ dextran. The activities of GSdGBd5R and GSdGBd4RL saturated with dextran were 35–38 s^−1^, or around 10% lower than GSdGBd6R. Furthermore, the activities of GSdGBd4RS and ΔNGBd saturated with dextran were greatly decreased, reaching 18–20 s^−1^ or ~50% of GSdGBd6R. Finally, the dextran‐saturated activities of GSdGBd3R and DCP were further decreased to as low as 6 s^−1^ or 15% of GSdGBd6R. Based on these results, we then categorized the enzymes into three groups: proteins with higher activity (i.e., GSdGBd6R, GSdGBd5R, and GSdGBd4RL), proteins with moderate activity (i.e., GSdGBd4RS and ΔNGBd) and proteins with lower activity (i.e., GSdGBd3R and DCP). This result indicates that GBd being longer than GBd4R is essential to retain glucosyl transfer function. Next, the reduced activity of DCP suggests that the orientation of GBd to GSd also regulates the glucosyl transfer to dextran. Finally, the N‐terminal GBd is necessary for highly efficient glucosyl transfer.

Interestingly, dextran showed slight substrate inhibitions for GSdGBd5R, GSdGBd4RL, GSdGBd4RS, and DCP. For this reason, ordinary Michaelis–Menten kinetics could not be applied to glucosyl transfer reactions involving dextran. Thus, we considered the dextran concentrations at which the activity is half of the apparent maximum activity as a sort of Michaelis constant. Such values for GSdGBd6R, GSdGBd5R, GSdGBd4RL, and DCP were all lower than 5 μg·mL^−1^ dextran. However, we also noted that these concentrations increased from GSdGBd4RS to GSdGBd3R. Overall, these results are generally consistent with our previous report, which found that the length of GBd correlates with the affinity of GBd for dextran [31]. Interestingly, the concentration of ΔNGBd was higher than the corresponding concentrations of GSdGBd5R, GSdGBd4RL, and GSdGBd4RS. ΔNGBd‐bound dextran may not be accessible to the catalytic domain since ΔNGBd may not be properly folded, as discussed below.

### Dextran binding

**Fig. 4:**
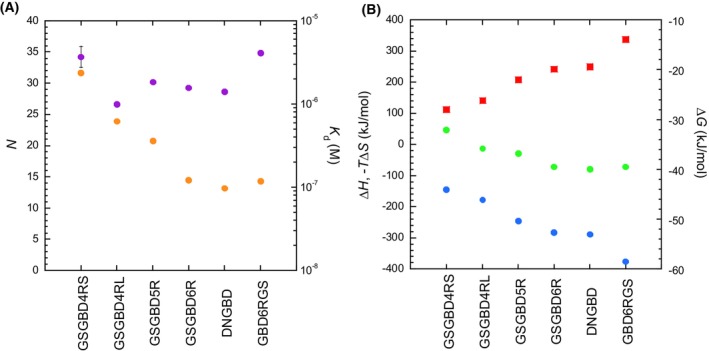
Isothermal titration calorimetry between glucosyltransferase (GTF)‐I and dextran. Shown are: (A) Dissociation constant (orange circles) and the number of bound glucose units (purple circles). (B) Thermodynamic parameters, including Δ*H* (enthalpy change) (blue circles), Δ*G* (Gibbs energy change) (green circles) and − *T*Δ*S* (entropy change) (red squares). Parameters are shown for GSdGBd6R, GSdGBd5R, GSdGBd4RL, GSdGBd4RS, DCP, and ΔNGBd. Furthermore, the binding of GSdGBd3R was too weak to permit determination of its parameters, and no heat signal was observed for GSdGBd2R or GSd. Data represent the mean ± standard error from independent experiments.

Our previous ITC measurement of the dextran‐binding capacity of truncated GBds (i.e., GBd4RS, GBd4RL, GBd5R, and GBd6R) suggested that the binding affinities, heats, and stoichiometries (Glc/GBd) (i.e., *K*_a_, Δ*H* and *n*) generally increase with an increasing number of repeated units [31]. Identically to our previous ITC analysis, here we used ITC to evaluate the dextran‐binding of truncated and permutated glucansucrase enzymes. First, neither GSdGBd2R nor GSd showed any binding heat for dextran (Fig. S3), indicating that neither enzyme bound to dextran. In contrast, GSdGBd3R exhibited exothermic heat of binding to dextran (Fig. S3). However, its titration curve was very gradual, suggesting that the affinity was very low. Accordingly, curve fitting for this enzyme did not yield quantitative parameters. Conversely, we also observed significant exothermic heats of binding to dextran for GSdGBd6R, GSdGBd5R, GSdGBd4RL, GSdGBd4RS, DCP, and ΔNGBd (Fig. S3), and their titration curves were able to be quantitatively fitted to a binding model that assumed that glucansucrase forms a 1 : 1 stoichiometric complex over *n* glucose units [31]. The thermodynamic parameters for these mutant enzymes are summarized in Fig. 4 and Table 1. Consistent with previous findings [31], the *K*_d_ and Δ*H* of the GBd‐truncated series were strongly correlated with GBd protein length (i.e., the number of amino acid residues) and followed the series GSdGBd6R < GSdGBd5R < GSdGBd4RL < GSdGBd4RS. In contrast, when *n* (i.e., the number of binding units of glucose) equaled 27–34 units, we found that the binding stoichiometry of glucose units showed no correlation with the number of amino acid residues present. This is notably different from the linear relation between *n* values from 18 to 35 and the number of amino acid residues in the GBd region [31]. This result suggests that the binding stoichiometries of glucose units are likely governed by GSd but not by GBd. Thus, the spatial distance intervals of glucansucrase on the dextran chain are clearly related to the size of GSd. This is consistent with the structural insight that there is a bending motion of the hinge region between GSd and GBd [15, 30].

**Table 1:** Thermodynamic parameters^a^ of glucansucrase‐mutant proteins binding to dextran.

Protein	*n*	*K*_d_ (M)	Δ*H* (kJ·mol^−1^)	Δ*G* (kJ·mol^−1^)	−*Τ*Δ*S* (kJ·mol^−1^)
GSd^b^					
GSdGBd2R^b^					
GSdGBd3R^c^					
GSdGBd4RS	34.2 ± 1.7	(2.36 ± 0.17) × 10^−6^	−145 ± 8	−32.1 ± 0.2	−113 ± 8
GSdGBd4RL	26.6 ± 0.12	(6.21 ± 0.28) × 10^−7^	−177 ± 2	−35.8 ± 0.2	−141 ± 2
GSdGBd5R	30.2 ± 0.17	(3.60 ± 0.21) × 10^−7^	−245 ± 3	−36.8 ± 0.2	−208 ± 3
GSdGBd6R	29.3 ± 0.13	(1.21 ± 0.07) × 10^−7^	−282 ± 3	−39.5 ± 0.2	−242 ± 3
DCP	34.8 ± 0.10	(1.18 ± 0.05) × 10^−7^	−376 ± 2	−39.5 ± 0.1	−337 ± 2
ΔNGBd	28.6 ± 0.16	(9.71 ± 0.74) × 10^−8^	−289 ± 3	−40.0 ± 0.2	−249 ± 3

^a^ The thermodynamic parameters characterizing the binding of glucansucrase and dextran were calculated from ITC data as a function of glucose according to a previously published protocol [31].

^b^ No heat production was observed for the titration of dextran with GSd and GSdGBd2R (Fig. S2).

^c^ The heat was produced during the binding of GSdGBd3R to dextran, but the nonlinear least‐squares curve fitting was incorrectly estimated due to gradual decreases in the heat curve caused by weak binding (Fig. S2). The numbers of experiments were as follows: 6 for GSdGBd4RS, 3 for GSdGBd4RL, 2 for GSdGBd5R, 4 for GSdGBd6R, 2 for DCP, and 3 for ΔNGBd. The values represent the mean ± standard error.

Relative to GSdGBd6R, the *K*_d_ and Δ*G* of DCP were similar, but we observed a decrease in Δ*H* and an increase in −*T*Δ*S* and *n*. These parameters are similar to those of GBd6R, a full‐length GBd without GSd [31]. Moreover, the GBd in DCP may bind to dextran just as GSd‐free GBd does. In contrast, the *K*_d_, Δ*H*, and *n* of ΔNGBd were approximately equal to those of GSdGBd6R. Taken together, these findings suggest that the N‐terminal GBd probably does not participate in dextran binding.

### CD analysis

**Fig. 5:**
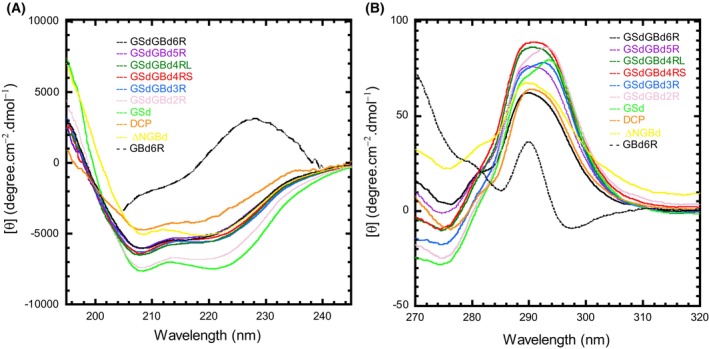
Circular dichroism (CD) spectra of glucosyltransferase (GTF)‐I proteins. Shown are: (A) Far‐UV spectra. (B) Near‐UV spectra. Spectra are shown for GSdGBd6R (black), GSdGBd5R (purple), GSdGBd4RL (green), GSdGBd4RS (red), GSdGBd3R (blue), GSdGBd2R (pink), GSd (light green), DCP (orange), and ΔNGBd (yellow). All measurements were recorded in 10 mM K‐phosphate, pH 6.8 at 20 °C. The black dashed line represents GBd6R. The far and near‐UV CD measurements were each repeated once, and similar spectra were obtained.

Next, we examined structural differences among truncated enzymes using CD (Fig. 5). The far‐UV–CD spectra of all enzymes revealed two minimum values at 208 and 222 nm. These are characteristic of α‐helices‐containing globular proteins and are in good agreement with the (β/α)_8_ barrel structure of the catalytic domain of glucansucrase [10, 11, 12, 13, 14, 15, 40]. Moreover, since the far‐UV–CD spectrum of GBd alone showed a positive value between 220 and 240 nm (Fig. 5A) [31, 40], the value at 222 nm may be correlated with GBd content. Therefore, the relatively high values recorded at 222 nm for GSdGBd6R, GSdGBd5R, GSdGBd4RL, GSdGBd4RS, GSdGBd3R, DCP, and ΔNGBd suggest that each contains GBd. Remarkably, GSdGBd6R, GSdGBd5R, GSdGBd4RL, GSdGBd4RS, and GSdGBd3R exhibited similar spectra, but DCP and ΔNGBd did not and showed more idiosyncratic spectra. In addition, the DCP showed lower values at both 208 and 222 nm than GSdGBd6R. Since the summation of the spectra of GSd and GBd generates a similar spectral profile as DCP (data not shown), each GSd and GBd in DCP probably fold independently. Furthermore, relative to GSdGBd6R, the molar ellipticity of ΔNGBd at 222 nm remained unchanged, but the reading at 208 nm had decreased. Thus, the deletion of the N‐terminal GBd may induce a conformational change in GSd. On the contrary, relatively low values at 222 nm in GSdGBd2R and GSd suggest low unfolding or low GBd content overall. These findings are consistent with the inability of GSdGBd2R and GSd to bind dextran, as described above.

The near‐UV–CD spectra of all enzymes showed broad positive bands around 290 nm (Fig. 5B). Moreover, the molar ellipticity of GBd6R peaked at 290 nm, while that of GSd peaked at 296 nm and displayed a shoulder at 290 nm. With respect to the peak and shoulder, the spectra of GSdGBd2R and GSdGBd3R were very similar to those of GSd. In contrast, the broad bands of GSdGBd6R, DCP, and ΔNGBd showed a maximum value around 290 nm, while those of GSdGBd5R, GSdGBd4RL, and GSdGBd4RS showed a swollen peak around 285–295 nm. Taken together, our results show that molar ellipticity around 290 nm and 296 nm somehow characterize the environment of the aromatic residues in GBd and GSd.

### Thermal stability

**Fig. 6:**
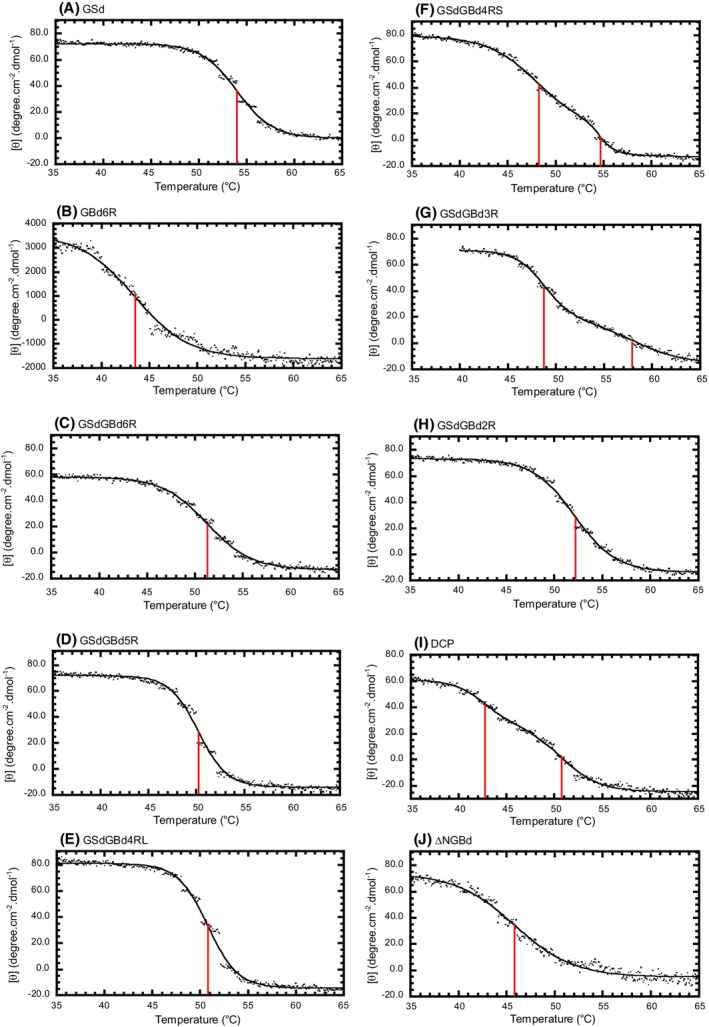
Thermal unfolding of glucosyltransferase (GTF)‐I proteins. Temperature‐induced changes in ellipticity were monitored at 292 nm at a speed of 1 °C·min^−1^. Black lines were fitted to single‐ or double‐transition models. Red lines indicate the obtained transition temperatures (*T*_m_). Results are shown for: (A) GSd, (B) GBd6R, (C) GSdGBd6R, (D) GSdGBd5R, (E) GSdGBd4RL, (F) GSdGBd4RS, (G) GSdGBd3R, (H) GSdGBd2R, (I) DCP, and (J) ΔNGBd. Near‐UV CD spectra on increasing temperature (Fig. S4) were independently measured, and data analysis from Fig. S4 yielded similar results.

The far‐UV–CD values of glucansucrases did not show monotonic changes with increasing temperature since the increase in the negative CD value of GSd was offset by a decrease in the positive CD value of GBd (data not shown). In contrast, near‐UV–CD spectra showed a monotonic decrease with increasing temperature (Fig. S4). Thus, near‐UV signals (i.e., molar ellipticities at 292 nm) rather than far‐UV signals were monitored as the temperature increased from 20 °C to 80 °C to evaluate protein thermal stability and unfolding (Fig. 6, Table 2). Moreover, GSd and GBd6R showed a single transition at *T*_m_ values of 54.0 °C and 43.5 °C, respectively (Fig. 6A,B). Overall, our findings suggest that GBd is much less stable than GSd. Next, we found that the two‐domain proteins GSdGBd6R, GSdGBd5R, and GSdGBd4RL showed single thermal unfolding transitions at 51.3 °C, 50.2 °C, and 50.8 °C, respectively (Fig. 6C–E). In these proteins, GSd and GBd are likely to unfold in a cooperative manner, with GSd stabilizing GBd and vice versa. Meanwhile, the molar ellipticities of GSdGBd4RS, GSdGBd3R, and DCP decreased with increasing temperature via a double transition (Fig. 6F,G,I). Next, the *T*_m1_/*T*_m2_ values of GSdGBd4RS, GSdGBd3R, and DCP were estimated to be 48.1/54.6 °C, 48.8/58.0 °C, and 42.7/50.7 °C, respectively. These three proteins appear to unfold in an incomplete yet cooperative manner. For example, GSdGBd2R unfolded while exhibiting a single transition at 52.1 °C (Fig. 6H), since the two‐repeat regions of GBd may be too small to fold as GBd does, but a shorter GBd can slightly affect the stability of GSd. Overall, thermal unfolding experiments indicated that whether the length of GBd was greater than that of GBd4RS is essential for cooperative unfolding. In addition, the *T*_m_ value of ΔNGBd decreased to 45.8 °C from 51.3 °C of GSdGBd6R (Fig. 6J); accordingly, ΔNGBd was less stabilized relative to GSdGBd6R. Taken together, these results demonstrate that the N‐terminal GBd plays an important role in glucansucrase folding.

**Table 2:** Transition temperatures^a^ of the thermal unfolding of glucansucrase‐mutant proteins.

Protein	Transition^b^	*T*_m1_ (°C)	*T*_m2_ (°C)
GSd	Single	54.0	
GBd6R	Single	43.5	
GSdGBd6R	Single	51.3	
GSdGBd5R	Single	50.2	
GSdGBd4RL	Single	50.8	
GSdGBd4RS	Double	**48.1** ^b^	54.6
GSdGBd3R	Double	**48.8** ^b^	58.0
GSdGBd2R	Single	52.1	
DCP	Double	42.7	**50.7** ^b^
ΔNGBd	Single	45.8	

^a^ Transition temperatures (*T*_m_s) were calculated from the temperature dependence of the CD ellipticities (Fig. 6) as described in the Methods section.

^b^ Bold numbers show the main transition of the protein.

## Discussion

The importance of interdomain allostery for glucansucrase function has been proposed by previous studies based on observed crystal structures as well as mutation studies [2, 15, 30]. In this study, we focused on how GBd affects glucosyl transfer of dextran. To do so, a series of mutant proteins were prepared, after which we evaluated the unfolding cooperativities of GS and related mutants by monitoring protein CD spectra, monitoring thermal stability in addition to glucosyl transfer activity and dextran‐binding affinity. This approach led us to discuss how the interdomain allostery between GSd and GBd may affect glucansucrase function.

The truncation of GBd has a major impact on the maximum rate of glucosyl transfers involving dextran. Overall, the activities of GSdGBd6R, GSdGBd5R, and GSdGBd4RL exhibited similar high activities to each other. However, GSdGBd4RS and GSdGBd3R showed markedly reduced activities, and GSd and GSdGBd2R showed no transfer activity (Fig. 3). This result suggests that the length of GBd, which is larger than that of GBd4R, is essential for achieving highly efficient glucosyl transfer to dextran. On the contrary, as consistent with our previous report [31], the binding affinity to dextran almost linearly increases with increasing GBd length (Fig. 4A). Furthermore, as per the results of our thermal unfolding study (Fig. 6, Table 2), all mutant proteins of GSd‐linked to GBd were found to be less stable than GSd alone.

Next, we found that GSdGBd6R, GSdGBd5R, and GSdGBd4RL were thermally unfolded following a single transition, suggesting that there may be cooperative folding between GBd and GSd. GSdGBd4RS and GSdGBd3R were thermally unfolded with a two‐phase transition and were probably impairing cooperative folding. Since it has smaller GBd than any of the others, GSdGBd2R unfolded with a single transition (like GSd), but was somehow less stable than GSd. Taken together, our findings suggest that glucosyl transfer efficiency may be correlated with the degree of folding cooperativity between GBd and GSd rather than their affinity to dextran. In other words, the interdomain allostery between GSd and GBd may enhance glucosyl transfer activity to dextran in GSdGBd6R, GSdGBd5R, and GSdGBd4RL. Notably, large decreases in entropy upon dextran binding (Fig. 4) also provide suggestive characteristics for the allosteric conformational change of GBd.

A permutation between GSd and GBd was found to greatly reduce the glucosyl transfer activity to dextran (Fig. 3), but maintained sufficient sucrose‐hydrolysis activity and a high affinity for dextran. Accordingly, the proximity effect of the acceptor may not be sufficient for efficient glucosyl transfer to dextran. The permutation may interrupt the orientation of the acceptor dextran chain in GSd. Notably, DCP showed a far‐UV–CD spectrum that was significantly different from GSdGBd6R (Fig. 5). In addition, this was found to unfold through a two‐phase transition at lower and higher temperatures, which may correspond with the unfolding of GBd and GSd, respectively (Fig. 6I). Taken together, these results suggest that folding cooperativity is weakened in the DCP.

The N‐terminal region of GBd is the N‐terminal half of domain V of two distinct parts on the U‐shaped form of the polypeptide. The deletion of N‐terminal GBd sequences resulted in a decrease in activity of ~50% (Fig. 3), suggesting that the N‐terminal GBd part participates in the glucosyl transfer. On the contrary, the dextran‐binding affinity of ΔNGBd was found to be comparable to that of GSdGBd6R (Fig. 4). Thus, neither the N‐terminal GBd part nor the domain V functions as a dextran‐binding unit. Thus, since GSd and GBd in ΔNGBd maintained sucrose‐hydrolysis activity and the dextran‐binding affinity, respectively, both GSd and GBd are thought to fold independently in the ΔNGBd. However, the observed far‐UV–CD spectrum of ΔNGBd distinctly differed from that of GSdGBd6R (Fig. 5). In addition, the thermal unfolding temperature of ΔNGBd was found to have greatly shifted to lower temperatures (Fig. 6J, Table 2). Overall, these results suggest that the interdomain folding structure is disrupted in ΔNGBd relative to GSdGBd6R. Therefore, the associated reduction of transfer activity is probably caused by disruption of cooperative interdomain folding between GSd and GBd via the N‐terminal portion of GBd. Moreover, domain V may contribute to interdomain allostery between GSd and GBd. A previous structural and functional study of *Leuconostoc* DSR‐M glucansucrase showed that deletion of the N‐terminal region of domain V resulted in decreased molar mass of dextran polymer without affecting the ability of the protein to catalyze polymer synthesis [15]. Consequently, domain V may be associated with the processive mechanism by which a longer glucose polymer is produced [1, 6, 32].

Next, we note that the straight and bending models of the crystal structure of *Lactobacillus* GS demonstrated that domain V likely functions as a hinge between GSd and GBd [2, 15, 30]. Therefore, reduced activity caused by the deletion of the N‐terminal part of GBd may be ascribed to the loss of this hinge function, which serves to bring GBd closer to GSd. Although the dextran‐binding stoichiometry of GBd alone was strongly correlated with the length of GBd [31], the stoichiometries of mutant proteins of GSd‐linked GBd did not change regardless of the length of GBd. Thus, the size of GSd can also help determine the stoichiometry in GSd‐linked mutants. Moreover, the binding of glucansucrase to the dextran chain may induce the bending motion between GSd and GBd. However, the manner and degree to which this bending motion is accommodated by interdomain allostery deserve further investigation.

Here, we demonstrate significant thermally unfolding cooperativity between GSd and GBd, which suggests that they engage in interdomain folding cooperatively. Moreover, a correlation between the unfolding cooperativity and glucosyl transfer activity implies that there is interdomain allostery regarding the glucosyl transfer function. Further analysis of ΔNGBd then indicated that the N‐terminal part of domain V is responsible for interdomain allostery. Information on this type of interdomain allostery may facilitate the development of novel drugs against dental caries. Although drugs development for targeting the catalytic site of glucansucrase have been reported [41, 42, 43], drugs that bind the glucan‐binding domain itself may be promising candidates for the suppression of dental plaque formation. The sequence of the catalytic domain of ancestral glucansucrases is evolutionally inserted into the ancestral domain V in GBd, and by this mechanism glucansucrase acquired the U‐shaped fold that tightly connected GSd to GBd in the overall structure [11]. Therefore, the resulting U‐shaped fold may determine the degree of interdomain allostery and hence control the efficiency of the transfer function. Previous studies have proposed that this U‐shape fold evolved via a ‘permutation per duplication model’ [9, 10, 11, 44], and this evolutionary process may be a key mechanism of the acquisition of higher functions.

## Conflict of interest

The authors declare no conflict of interest.

## Author contributions

HK and TK designed the research. HK, TS, and YM contributed to investigation and data analysis. TK supervised the project and provided funding for the project. HS contributed to project administration. JT and HS aided in plasmid construction, protein expression, and purification. HK wrote the manuscript. All authors have approved the final version of the manuscript for publication.

## Peer review

The peer review history for this article is available at https://www.webofscience.com/api/gateway/wos/peer‐review/10.1002/1873‐3468.70128.

## Supporting information


**Fig. S1.** Plasmid construction of GTF‐I mutant proteins.
**Fig. S2.** SDS/PAGE of purified GTF‐I mutant proteins.
**Fig. S3.** Gallery of ITC raw data and titration curve.
**Fig. S4.** Near‐UV CD Spectra changes on increasing temperature.

## Data Availability

The authors confirm that the data supporting the findings of this study are available within the main text and the Supporting Information. The data that support the findings of this study are available from the corresponding authors upon reasonable request and on Dryad at https://doi.org/10.5061/dryad.bcc2fqzr5.
