# Supplementary material for: Functional role and folding properties of the glucan‐binding domain of oral bacterial glucansucrase
Source: FEBS Lett. 2025 Aug 2;599(16):2388–402. doi: 10.1002/1873-3468.70128 (PMC12375890; doi:10.1002/1873-3468.70128)
Supplement: Supplementary file 1 — Fig. S1. Plasmid construction of GTF‐I mutant proteins. Fig. S2. SDS/PAGE of purified GTF‐I mutant proteins. Fig. S3. Gallery of ITC raw data and titration curve. Fig. S4. Near‐UV CD Spectra changes on increasing temperature. [file FEB2-599-2388-s001.pdf]

Supplemental figures

Figure S1. Plasmid construction of GTF-I mutant proteins.

(A) Construction of plasmids encoding the C-terminal glucan-binding domain-truncated GSdGBd6R (GSdGBd5R, GSdGBd4RL, GSdGBd3R, GSdGBd2R) and the N-terminal glucan-binding domain (amino acid 85–212)-truncated GSdGBd6R (ΔNGBd). The plasmids pGS-GBD5R, pGS-GBD4RL, pGS-GBD3R and pGS-GBD2R were constructed from pGBD5R, pGBD4RL, pGBD3R, pGBD2R and pGS-GBD6R. (B) Construction of plasmids encoding the domain-circularly permuted protein (DCP). The plasmid pGBD6R-GS was constructed from pGS and pAB5. The details were described in Martials and Methods.

Figure S2. SDS-PAGE of purified GTF-I mutant proteins.

M: Molecular weight markers, 1:GSdGBd6R, 2: GSdGBd5R, 3: GSdGBd4RL, 4: GSdGBd4RS, 5: GSdGBd3R, 6: GSdGBd2R, 7: GSd, 8:DCP, 9: ΔNGBd.

Figure S3. Gallery of ITC raw data and titration curve.

The accumulated heat curves of GSdGBd6R, GSdGBd5R, GSdGBd4RL, GSdGBd4RS, DCP and ΔNGBd were fitted with 1:1 binding model to a glucose unit. The fitting of heat curves of GSdGBd3R were failed not to obtain the parameters. No binding heat signal of GSdGBd2R and GSd was observed.

Figure S4. Near UV CD Spectra changes on increasing temperature.

The spectra were recorded from 36 to 66 °C at intervals of 2 °C.

Fig. S1

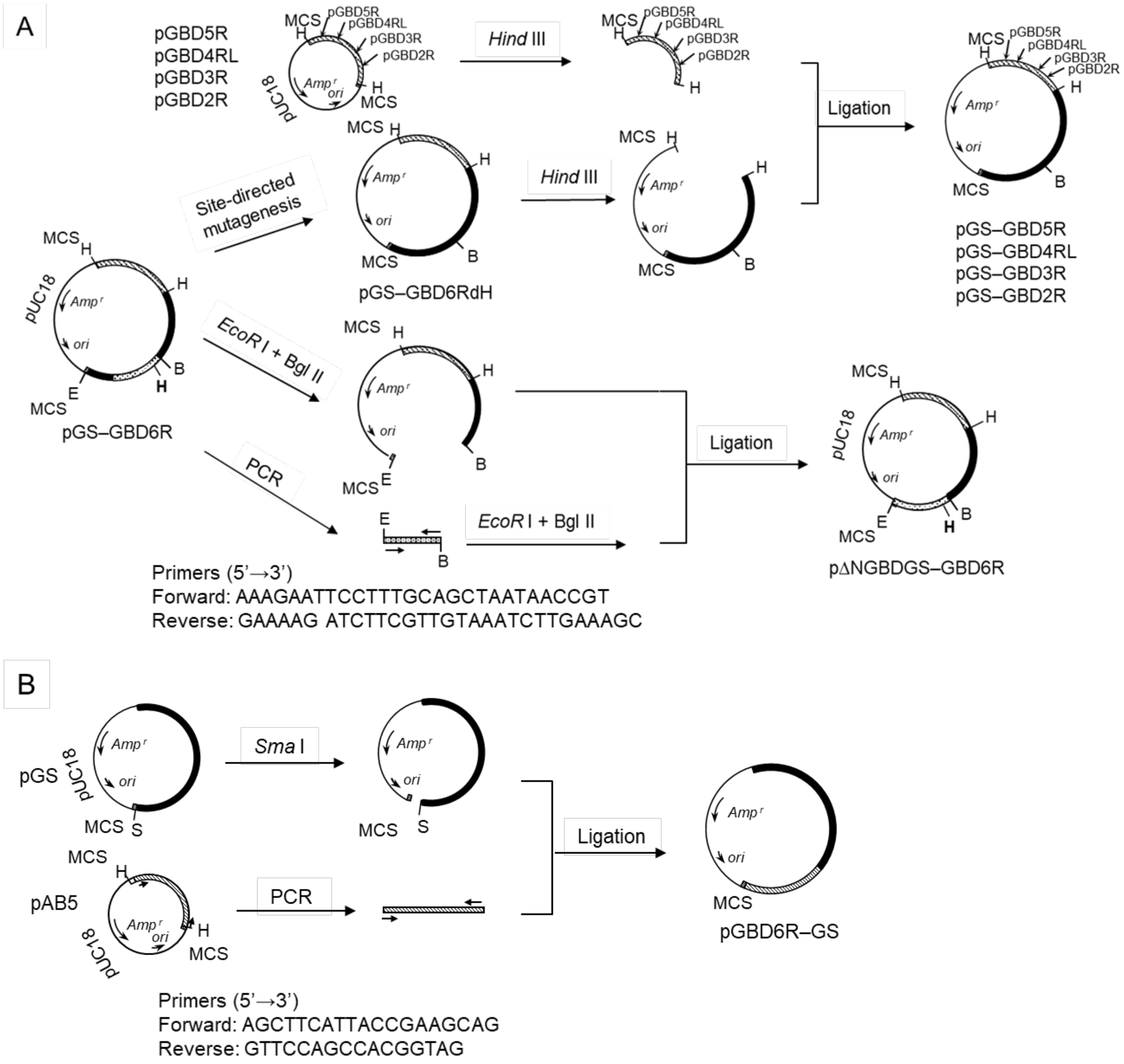
A
pGBD5R
pGBD4RL
pGBD3R
pGBD2R
MCS
H
pUC18
Amp r
ori
Hind III
Ligation
Site-directed mutagenesis
pGS–GBD6RdH
B
E
pGS–GBD6R
EcoR I + Bgl II
PCR
pGS–GBD5R
pGS–GBD4RL
pGS–GBD3R
pGS–GBD2R
pΔNGBDGS–GBD6R
Primers (5'→3')
Forward: AAAGAATTCCTTTGCAGCTAATAACCGT
Reverse: GAAAAG ATCTTCGTTGTAAATCTTGAAAGC
B
pGS
Sma I
S
pAB5
pGBD6R–GS
Primers (5'→3')
Forward: AGCTTCATTACCGAAGCAG
Reverse: GTTCCAGCCACGGTAG

Fig. S2

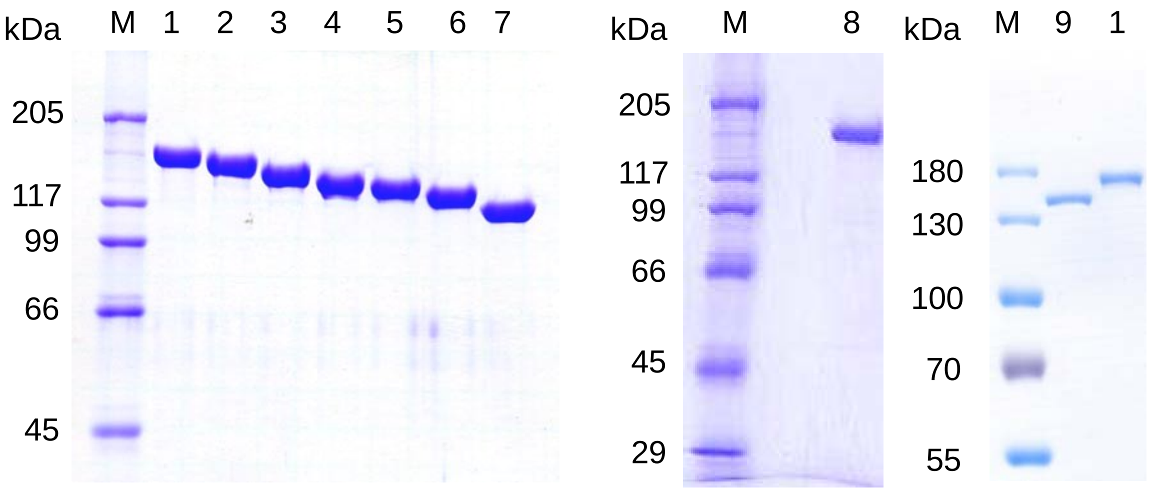
kDa
M 1 2 3 4 5 6 7
205
117
99
66
45
kDa
M 8
205
117
99
66
45
29
kDa
M 9 1
180
130
100
70
55

Fig. S3

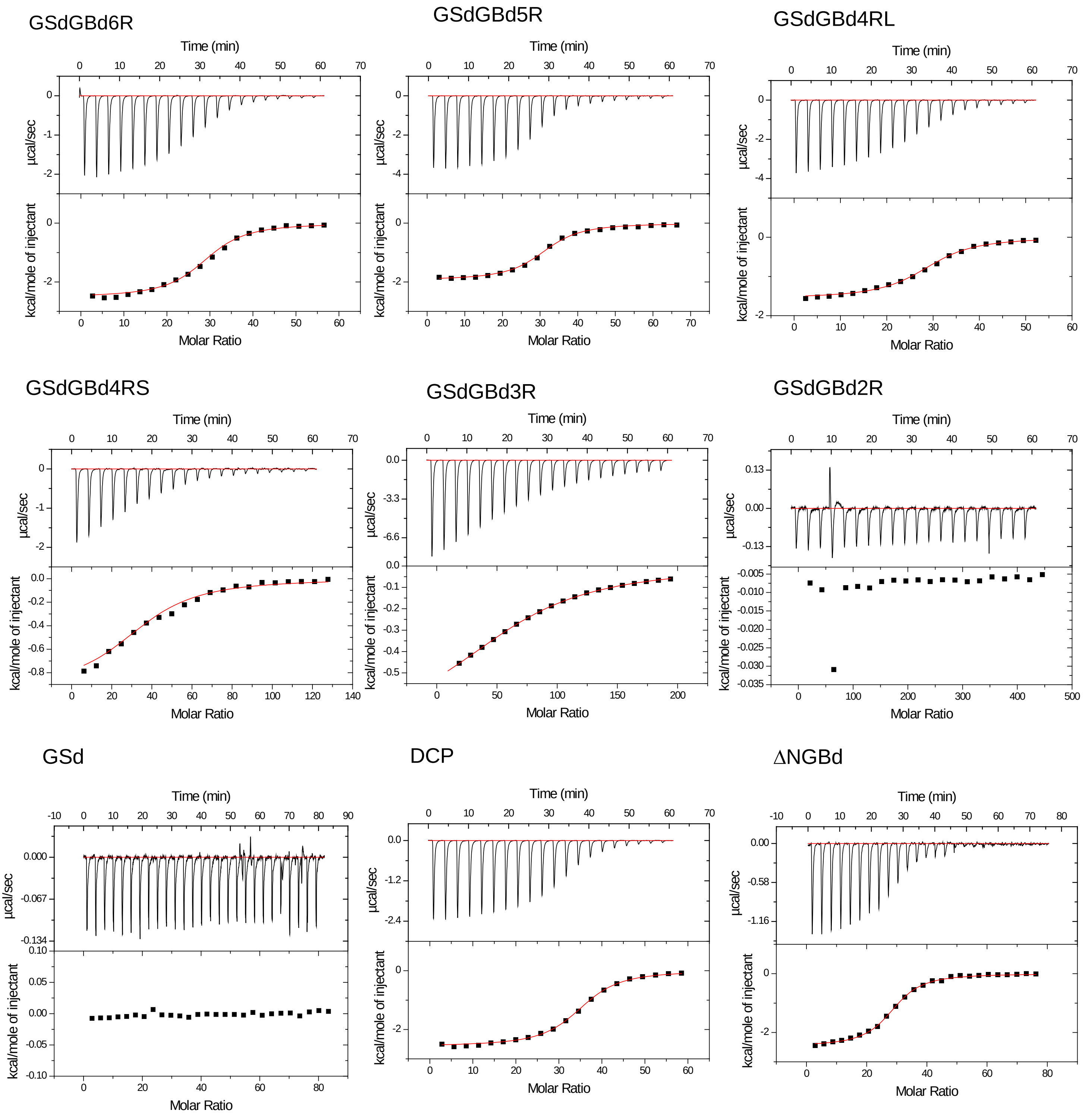
GSdGBd6R
GSdGBd5R
GSdGBd4RL
GSdGBd4RS
GSdGBd3R
GSdGBd2R
GSd
DCP
ΔNGBd
Time (min)
µcal/sec
kcal/mole of injectant
Molar Ratio

Fig. S4

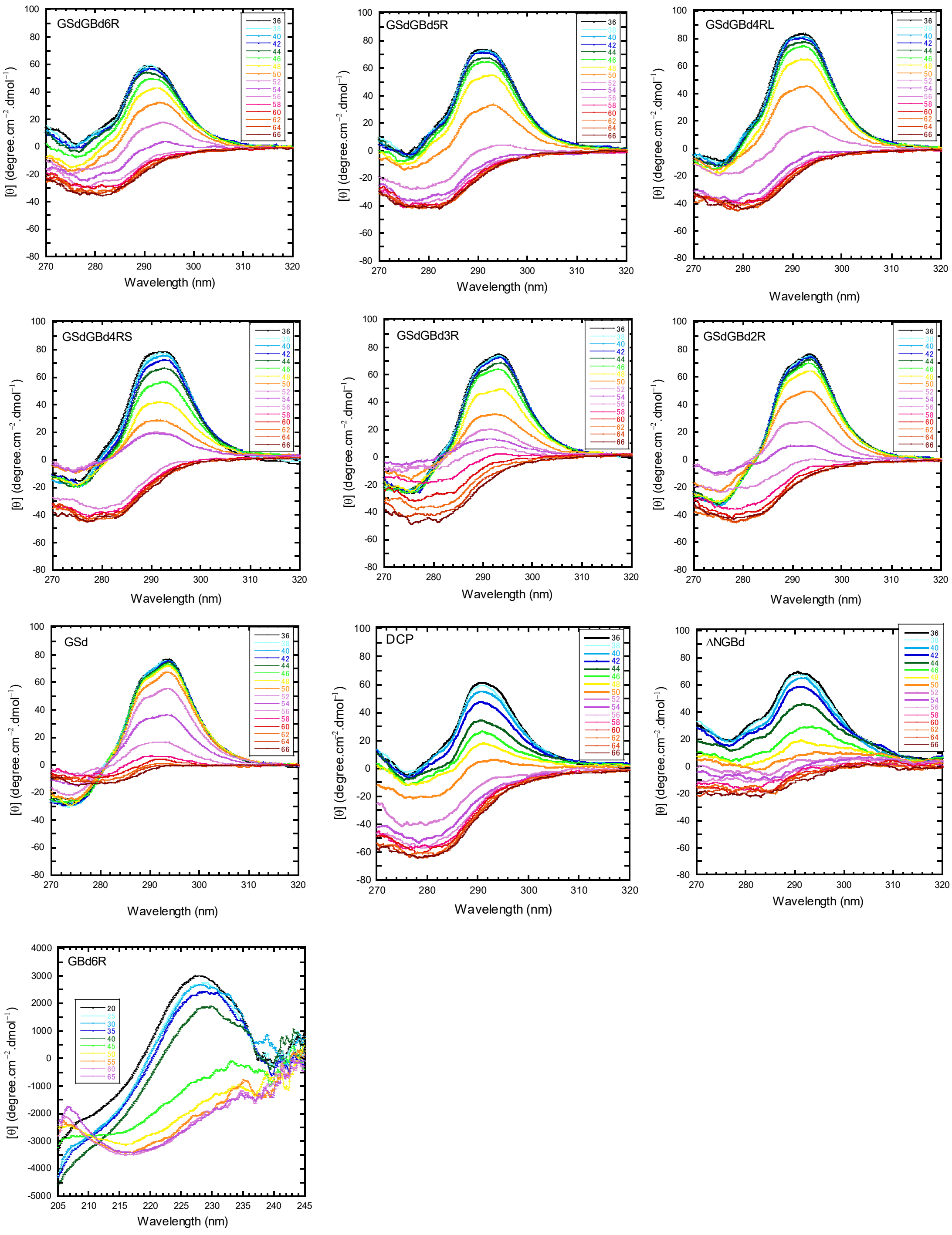
GSdGBd6R
GSdGBd5R
GSdGBd4RL
GSdGBd4RS
GSdGBd3R
GSdGBd2R
GSd
DCP
ΔNGBd
GBd6R
[θ] (degree.cm⁻².dmol⁻¹)
Wavelength (nm)
